# Phelan–McDermid syndrome in a Chinese pediatric patient: A case report – new heterozygous mutations lead to PMS

**DOI:** 10.1097/MD.0000000000044114

**Published:** 2025-09-05

**Authors:** Shuangzhu Lin, XiaoYu Sun, Ying Zhou, ZiXuan Ding, Kai Jiang, JinHua Feng

**Affiliations:** a College of Traditional Chinese Medicine, Changchun University of Chinese Medicine, Changchun, Jilin Province, China; b Diagnosis and Treatment Center for Children, The Affiliated Hospital to Changchun University of Chinese Medicine, Changchun, Jilin Province, China; c College of Acupuncture and Massage,Changchun University of Chinese Medicine, Changchun, Jilin Province, China.

**Keywords:** case report, developmental delay, genetic conditions, systematic rehabilitation

## Abstract

**Rationale:**

Phelan–McDermid syndrome, also known as chromosome 22q13.3 deletion syndrome, is a genetic disorder primarily caused by a chromosome 22q13.3 deletion or mutation. The primary clinical manifestations include intellectual disability, delayed language development, behavioral delays, hypotonia, autism spectrum disorder, mild deformities, and epilepsy. The clinical symptoms of this disease are associated with chromosomal deletions, and mild cases may be easily misdiagnosed as autism spectrum disorder.

**Patient concerns:**

A 3-year-old girl was admitted for “chromosomal abnormality (heterozygous deletion) and developmental delay.” After admission, we found that the child’s overall growth retardation (mainly language and movement) and accompanied by obvious social disorders, but the muscle strength and muscle tension were basically normal; brain magnetic resonance imaging and electroencephalography were not obvious abnormalities. Gene copy number variation analysis showed that there was a new pathogenic heterozygous deletion of 1.21 Mbp in the chromosome 22:50014294–51220722 region, and the genomes of both parents were wild-type.

**Diagnoses:**

Combined with the clinical manifestations of the child, the child was finally diagnosed with mild Phelan–McDermid syndrome.

**Interventions:**

The children received systematic rehabilitation treatment.

**Outcomes:**

Her language, social, and motor abilities were significantly improved.

**Lessons:**

Phelan–McDermid syndrome may be easily misdiagnosed as autism spectrum disorder. Our report enriches the clinical phenotype of Phelan–McDermid syndrome and provides a realistic and reliable basis for clinicians.

## 1. Introduction

Phelan–McDermid syndrome (PMS) is characterized by developmental delay, hypotonia, speech loss or severe speech delay, typical behavioral abnormalities, mild physical deformities, seizures, and sleep disorders. Intellectual disability is a common symptom of PMS.^[1]^ The main etiology of this syndrome is attributable to the deletion or mutation of chromosome 22q13.3, including the *SHANK3* gene. The neurological manifestations are largely the result of *SHANK3* haploinsufficiency, due to loss of gene function on chromosome 22.^[2]^

*SHANK3* consists of 22 exons and is predominantly expressed in neurons, especially at synaptic sites.^[3]^ The gene is primarily responsible for encoding the main scaffold

proteins at excitatory synapses, coordinating and aggregating signal molecules, and establishing scaffolds for the proper arrangement of neurotransmitter receptors, which can promote the development and maturation of excitatory synapses.^[4]^ In an experimental study, Peça et al.^[5]^ reported a decreased number of functional synapses and diminished postsynaptic responses in *SHANK3*-deficient mice, which also exhibited repetitive self-injurious behaviors and impairments in social interaction.

## 2. Case presentation

A 3-year-old girl was first observed with delayed language development during a routine checkup at the age of 1 year and 4 months. The specific manifestations included unclear speech and the inability to articulate single words; however, these symptoms did not prompt concern or intervention from her parents. The child was admitted to the local “rehabilitation institution” for treatment at the age of 2 years and 2 months. The child’s speech ability gradually improved over time, but the progress remained limited. On July 23, she was admitted to the “Changchun Hospital of Integrated Traditional Chinese and Western Medicine” and diagnosed with “developmental delay” following examination. During this period, the patient underwent rehabilitation therapy upon request. In August of the same year, she was admitted to the outpatient clinic of our hospital with the chief complaint of “chromosomal abnormality (heterozygous deletion) and developmental delay.” Current symptoms: The child is alert but exhibits a poor response to verbal cues and maintains only brief eye contact. She demonstrates limited ability to follow instructions. Although she can walk independently, her gait is unsteady. She is unable to run and can navigate stairs only with the aid ofa handrail.

After admission, we inquired in detail regarding the child’s birth and growth history. The child was born full-term as the first pregnancy and delivery, with a birth weight of 3.0 kg and a birth height of 50 cm. The birth was uncomplicated, with no history of asphyxia or resuscitation. The mother was healthy during pregnancy, denied any history of special medication or exposure to radioactive substances, and had no abnormalities in the perinatal period. The child was able to sit independently at 8 months of age, stand with support at 1 year, and walk unaided at 18 months. Language milestones included speaking single words at 18 months and forming phrases by 2 years and 6 months of age.

The patient remained healthy. He denied any history of infectious diseases, such as measles, chickenpox, mumps, and whooping cough, or surgical trauma. He denied any history of blood transfusions; however, his vaccination history was reported as up to date. He reported no known drug allergies but had a documented history of milk allergy. His parents were reportedly healthy, and no family history of genetic diseases or developmental delays was noted.

A detailed physical examination of the patient demonstrated a height of 95.0 cm and a weight of 11.9 kg. The patient was in good general condition with moderate nutritional status. Mental status was alert, although responses were slow. Both pupils are equally round and sensitive to light. No rashes, milk-coffee spots, or depigmentation spots were observed on the skin. Furthermore, examination of the palmar dermatoglyphics revealed no abnormalities. The thoracic spine was symmetrical and undeformed, and no abnormalities were observed on the cardiopulmonary examination. No tenderness or rebound tenderness was noted in the abdomen, liver, or spleen. No obvious abnormalities were observed on limb examination. Muscle strength and tone in the limbs were within normal limits. Even though physiological reflexes were present, no pathological reflexes were induced. Motor development assessment: Gross motor skills: The child can walk independently but exhibits an unsteady gait and requires support from a handrail when ascending and descending stairs.

Fine motor skills: demonstrates poor bilateral hand coordination, can grasp small objects between the thumb and forefinger, and is capable of stacking 3 blocks. Intellectual and language development: The child can speak in phrases but exhibits a poor response to being called by name, maintains brief eye contact during interactions, prefers solitary play, and has difficulty following verbal instructions.

**Figure 1.**
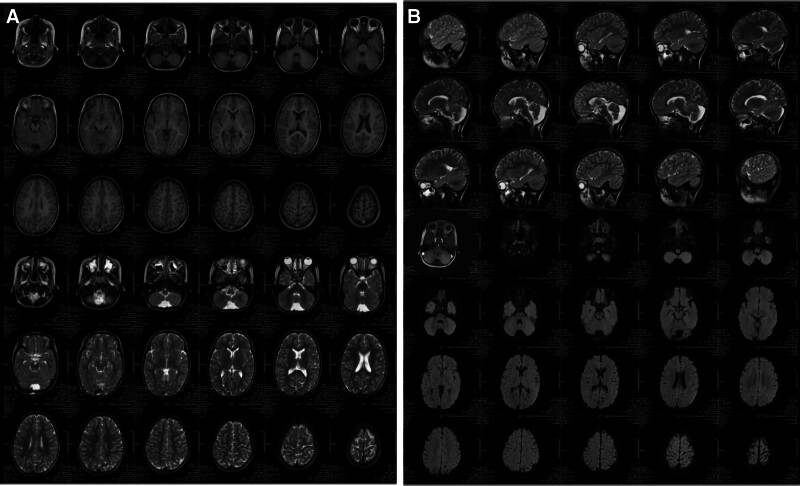
(A and B) Bilateral cerebral hemispheres are symmetrical, with no obvious abnormality in gray and white matter differentiation. No focal signal abnormalities are noted, and the size and shape of each ventricle and cistern are within the normal limits. The midline structures are centered. The morphology and signal intensity of subependymal regions, cerebellum, and brainstem exhibit no abnormalities. Sagittal view revealed no obvious abnormalities in the size and morphology of the pituitary gland. The great foramen of the occiput is enlarged. The bilateral maxillary and ethmoid sinuses exhibit thickened mucosa.

**Figure 2.**
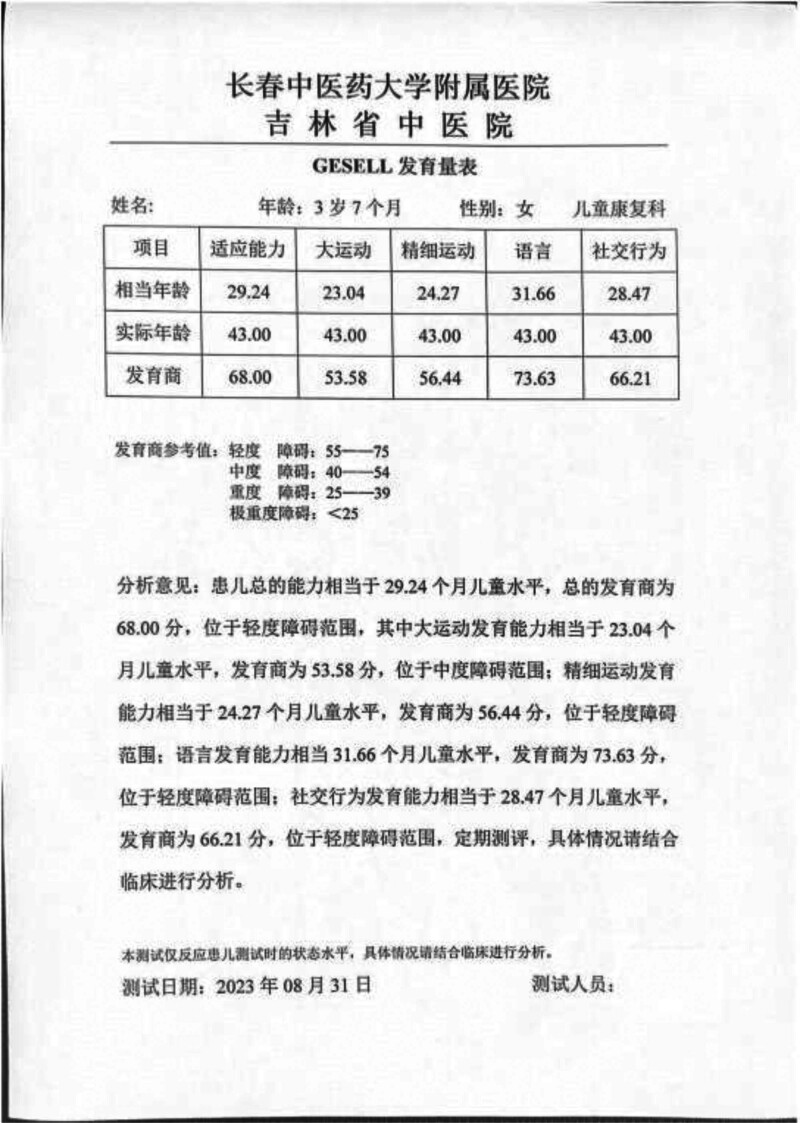
Gesell developmental assessment.

**Figure 3.**
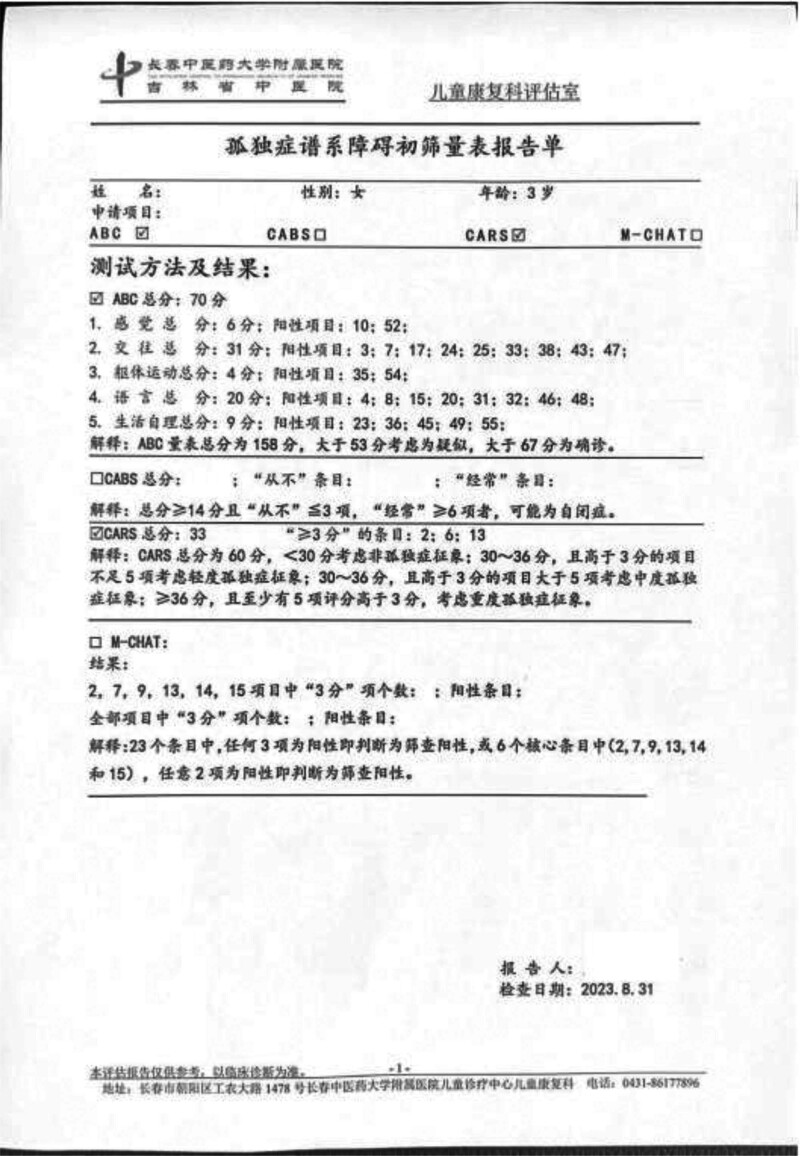
Autism behavior checklist scale score.and childhood autism rating scale score.

Laboratory tests revealed no abnormalities in the blood, urine, routine stool, thyroid function, liver and kidney function, electrolyte levels, blood glucose, cardiac enzymes, hematuria, genetics, and metabolism. Electrocardiogram findings were within normal limits. No abnormalities were observed on cardiac ultrasound, as well as in the digestive or urinary systems. Head MRI showed 1. Great occipital pool; 2. Paranasal sinus inflammation (Figs. 1A and B). Video electroencephalography (EEG) examination demonstrated no obvious abnormalities. The Ceschell developmental assessment identified mild developmental delays across the following domains: adaptive skills (68.00), gross motor skills (53.58), fine motor skills (56.44), language (73.63), and personal-social skills (66.21) (Fig. 2). The autism behavior checklist scale score was 70 points; meanwhile, the childhood autism rating scale score was 33 points, indicative of mild autistic traits (Fig. 3).

**Figure 4.**
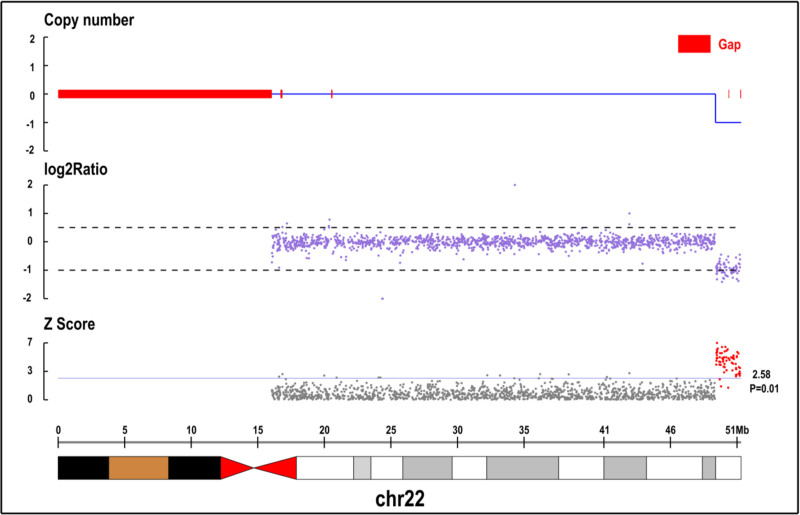
Patient, proband, heterozygous loss.

**Figure 5.**
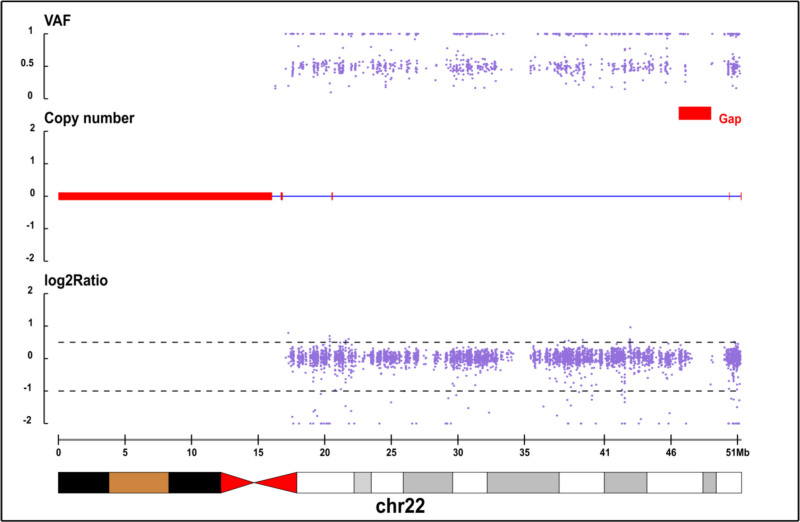
Proband’s father, normal, wild-type.

**Figure 6.**
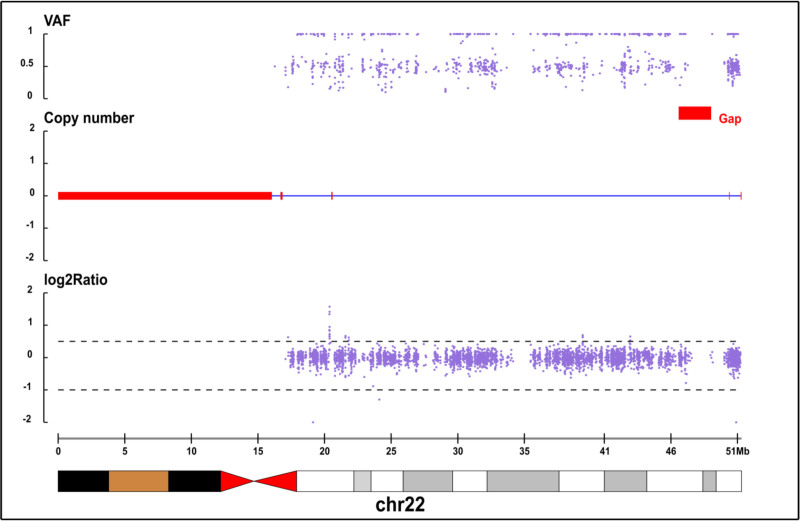
Proband’s mother, normal, wild-type.

Additionally, whole-exon examination revealed no abnormalities. Copy number variation (CNV) analysis revealed a 1.21 Mb heterozygous deletion (Seq [GRCH37] DEL (22) (q13.33 q13.33) | NC _ 000022.10: g.50014294 _ 5122772 DEL) at chr22:50014294-51220722 in the child (Fig. 4), while both parents exhibited wild- type alleles (Figs. 5 and 6). The mutation was considered pathogenic according to the American College of Medical Genetics and Genomics guidelines.

During the physical examination, we observed that the child exhibited a poor response to her name, limited eye contact with others, solitary play, and reduced ability to follow commands. The symptoms were consistent with the clinical presentation of speech and language developmental delay and autism in PMS.

The final diagnosis of this case was PMS, with targeted rehabilitation training provided to address motor developmental delays, language impairments, and social dysfunction. At present, the patient has completed 1 year of systematic rehabilitation training, and she can now run unaided, speak simple sentences, execute simple 2-step instructions, and draw simple pictures. Her language, motor, and social functioning have also improved significantly.

## 3. Discussion

Children with PMS present with global developmental delays associated with cognitive deficits, behavioral challenges, and features consistent with autism spectrum disorder (ASD), as well as mild dysmorphic facial characteristics. Some children may also demonstrate congenital renal abnormalities, as well as neurological manifestations, including abnormal motor patterns and seizures.^[6]^ However, the clinical prevalence of PMS remains unknown. Due to subtle physical deformities, vague clinical symptoms, and a lack of accurate clinical laboratory diagnoses, PMS often remains underrecognized in clinical practice.^[2]^

We noted that the patient underwent auxiliary speech training at a rehabilitation institution during the disease. However, during clinical treatment, the patient exhibited a poor response to her name, had limited eye contact with others, and could walk independently but with an unstable gait. She relied on the railings to climb the stairs. She exhibited poor hand coordination, preferred solitary play, and had difficulty following instructions. These symptoms are consistent with the main clinical features of PMS, such as language and speech deficits, delayed overall development, and normal-to-late growth.^[3]^ ASD is another characteristic feature of PMS. In clinical practice, children who exhibit poor responsiveness to their name, limited eye contact, impaired ability to follow commands, and difficulty engaging in typical social interactions often present with social and communication deficits, which are hallmark features of ASD.^[7]^ In addition, no other behavioral abnormalities or history of seizures was reported. Furthermore, no significant abnormalities were observed on video-EEG or cranial MRI. No significant head or facial malformations were observed upon physical examination.

Targeted rehabilitation training was performed for children with impaired motor development, language delays, and social disorders. Currently, the patient has completed a year of systematic rehabilitation training, resulting in remarkable improvements. Notably, her language, social, and motor skills have progressed; she is now able to speak simple sentences, follow basic 2-step instructions, and draw simple pictures.

The etiology of PMS is the deletion of the 22q13.3 gene segment, with *SHANK3* being the primary contributor to nervous system disorders, including PMS. *SHANK3* has various functions, including interaction with ion channel receptors, signaling molecules, cytoskeletal proteins, and regulation of dendritic spine maturation.^[8]^ *SHANK3* is highly sensitive to mutations, with alterations often resulting in significant neurological effects. Haploid deficiency is the main cause of neurological lesions and communication disorders.^[9]^ Animal studies have demonstrated that mice with *SHANK3* deficiencies exhibit varying degrees of synaptic dysfunction. The downregulated expression of *SHANK3* leads to a decrease not only in the number of dendrites but also in synaptic transmission.^[5]^ Breen et al.^[10]^ reported that *SHANK3* deficiency affects early developmental pathways and presynaptic and postsynaptic signal transduction.

Loss-of-function mutations in *SHANK3* predispose individuals to ASD symptoms, seizures, and EEG abnormalities. A recent genome-wide association study of CNV and developmental disorders, including ASD, indicated that *SHANK3* is the primary gene responsible for ASD.^[11]^ Betancur^[12]^ reported that *SHANK3* haploinsufficiency occurred in approximately 0.5% of patients with ASD. Soorya et al.^[13]^ studied 32 patients with *SHANK3* haploinsufficiency and reported that 84% met the ASD criteria. Moreover, Tess Levyet al.^[14]^ analyzed 98 patients with the 22q13.3 deletion syndrome and reported that 42% of them had epilepsy. The study demonstrated that patients with larger gene deletions exhibited a higher incidence of seizures. Lavanya^[15]^ reported that the highest incidence of epilepsy was observed in patients with *SHANK3* point mutations and deletions > 4 Mb. In a study involving 32 patients with PMS, Soorya et al.^[16]^ observed that 13% of the patients demonstrated EEG abnormalities without clinical seizures. Thus, epileptic discharges can be detected on EEG in patients with PMS regardless of the presence of seizures.

Currently, the diagnosis of PMS primarily relies on genetic testing. Chromosome microarray analysis can diagnose most patients with PMS and 22q13 CNV (>30 Kb) caused by simple chromosome deletion or rearrangement.^[17]^ Patients with PMS due to small microdeletions or duplications of *SHANK3* can be identified through deoxyribonucleic acid sequencing to prevent missed or misdiagnosis. Patients diagnosed by chromosome microarray analysis or CNV monitoring require high-resolution karyotyping to assess for the presence of the ring 22 chromosome.^[18,19]^

Key aspects of PMS management include early diagnosis, comprehensive treatment, dynamic tracking, and follow-up. Standardized pharmacological treatments and rehabilitation training for language, behavioral, and motor symptoms remain key areas requiring further investigation and optimization in the treatment of patients with PMS. Significant advancements in specific pharmacological agents and therapeutic approaches have been achieved in recent clinical trials. For instance, by intraperitoneally injecting insulin-like growth factor-1 into *SHANK3*-deficient mice, Bozdagi^[20]^ treated motor dysfunction by improving the conduction of α-amino-3- hydroxy-5-methyl-4-isoxazolepropionic acid receptor mediators. According to Qin et al.^[21]^ β-hydroxybutyric acid, the primary metabolite of the ketogenic diet, functions as an endogenous class I histone deacetylase inhibitor, which can restore the synaptic function of *SHANK3*-deficient mice by improving the histone acetylation level in prefrontal cortex neurons, thereby providing a potential treatment strategy for patients with PMS. Symptomatic treatment plays an important role in the clinical management of patients with PMS. For instance, Sethrisstad et al systematically treated a patient with intellectual disability and ASD due to 22q13.3 deficiency syndrome using antipsychotic medications such as aripiprazole, risperidone, and olanzapine. After 5 years of systemic treatment, the patient’s symptoms significantly improved.^[22]^ Moreover, Pasini^[23]^ treated an 18-year-old woman with PMS using a low dose of risperidone (1 mg).

## 4. Conclusion

We report a case of PMS in a child diagnosed through CNV analysis, revealing a novel pathogenic heterozygous deletion of 1.21 Mb at chr22:50014294-51220722. The main manifestations included poor response to name-calling, limited eye contact with others, impaired bilateral hand coordination, preference for solitary play, difficulty following commands, and an unsteady gait. After a year of systematic rehabilitation, the language, motor, and social functions of the patient significantly improved.After a year of systematic rehabilitation, the language, motor, and social functions of the patient significantly improved.Unfortunately, compared with children of the same age, our children are still lagging behind in all aspects of development, and their social barriers still exist. We will continue to pay attention to the development level of children, specify personalized rehabilitation programs, and promote the recovery of children’s condition.

The patient with PMS exhibited minimal overall clinical symptoms, and following thorough discussion with her guardian, experimental medication was not administered. In summary, we reported a case of PMS caused by a novel heterozygous deletion, whose symptoms were relatively mild and clinically the disease is easily confused with ASD. Through this case report, we hope to deepen our understanding of the clinical phenotype of children with PMS and provide a reference for clinical diagnosis and treatment, so that PMS can be identified and diagnosed in a timely manner, early intervention can be made, and the prognosis can be improved.

## Acknowledgments

We thank the patient and her family members for their contributions to this study.

## Author contributions

**Writing – original draft:** Shuangzhu Lin, Xiaoyu Sun, Ying Zhou, ZiXuan Ding.

**Writing – review & editing:** Shuangzhu Lin, Kai Jiang, Jinhua Feng.
